# What goes up must come down: insights from studies on descending controls acting on spinal pain processing

**DOI:** 10.1007/s00702-019-02077-x

**Published:** 2019-09-12

**Authors:** Stevie Lockwood, Anthony H. Dickenson

**Affiliations:** Department of Neuroscience, Physiology and Pharmacology, University College London, Gower St., London, WC1E6BT UK

**Keywords:** Diffuse noxious inhibitory controls (DNIC), Conditioned pain modulation (CPM), Descending controls, Noradrenaline, 5-hydroxytrptamine, Spinal cord

## Abstract

Descending controls link higher processing of noxious signals to modulation of spinal cord responses to their noxious inputs. It has become possible to study one key inhibitory system in animals and humans using one painful stimulus to attenuate another distant response and so eliciting diffuse noxious inhibitory controls (DNIC) or the human counterpart, conditioned pain modulation (CPM). Here, we discuss the neuronal pathways in both species, their pharmacology and examine changes in descending controls with a focus on osteoarthritis. We will also discuss the opposing descending facilitatory system. Strong parallels between DNIC and CPM emphasize the possibility of forward and reverse translation.

There is often a lack of clear correlation between the degree of peripheral damage, whether due to a trauma or disease, and the level of pain perceived. This may result from changes in a number of pain sensitising or pain modulating systems at peripheral and central levels (see Bannister et al. 2017a, b). Altered processing at peripheral levels will alter the nociceptive drive into the spinal cord where events, such as central sensitisation may emerge (Arendt-Nielsen et al. 2018). But if the spinal cord level of excitability alters then ascending messages to the brain will change and in turn, descending controls that arise from midbrain and brainstem regions will have a different impact on sensory processing in the spinal cord (Bannister and Dickenson 2016a, b). This review considers the ways in which descending pathways can modulate pain at spinal levels, their pharmacological basis and anatomical circuits. Attention will focus on the parallel animal and human data using Diffuse Noxious Inhibitory Controls (DNIC) whereby one noxious stimulus can inhibit activity in deep spinal cord neurones distant to the conditioning stimulus through descending controls (Le Bars et al. 1978a, b) and conditioned pain modulation (CPM) where one pain can inhibit another in humans (Yarnitsky 2015). Personalised medicine can use these systems to probe the integrity of descending pathways and target their pharmacology as has been used in neuropathic pains (Colloca et al. 2017). Here we also consider the roles of descending controls in models of osteoarthritis, a common pain condition which has not been examined in the same detail as neuropathic pain. We emphasise translational studies in both directions.

## Circuitry behind descending controls

Descending controls form a link between the brain and spinal cord and allow top down modulation of spinal processing of afferent inputs. Indeed, in normal conditions, in humans, there is a continual conversation between brain and spinal cord that correlates with the pain perceived (Stroman et al. 2018). Descending systems originate in the brainstem and midbrain yet are controlled by higher centres and form an endogenous modulating circuit. The periaqueductal grey, situated in the midbrain is a key area receiving inputs from forebrain areas and in turn modulates brainstem areas such as the rostroventromedial medulla (RVM) that then project to the spinal cord. Alongside the RVM, there are noradrenergic pathways to the spinal cord from the locus coeruleus (LC) (see Bannister and Dickenson 2016a, b). These pathways allow for higher centres to regulate pain at the first synapses in the pathways from the periphery to the CNS and it is noteworthy that psychological processes can modulate these systems. The best examples come from human imaging where placebo and nocebo, the former through endogenous opioid signalling, activate descending inhibitions and facilitations, respectively (Eippert et al. 2009a, b; Tinnermann et al. 2017). These studies fit well with ideas from preclinical research where the descending pathways exert bidirectional effects on pain processing. Seminal studies from Basbaum and Fields (1978, 1984) described On and Off cells, whereby their activity could be related to behavioural responses induced by heat. Off cells ceased firing just before the response, whereas On cells fired. This bidirectional action has been verified by many studies. Opioid analgesia is associated with the converse, switching Off cells on and inhibiting On cell activity with the RVM.

## Pharmacological profiles of inhibitions and facilitations

The identity of the transmitters in descending control systems has long been studied with indications of 5HT and NA playing an important role and as verified by the human placebo study, endogenous opioids have been characterised and indeed, On cells have opioid receptors on them. This allows On cells to be manipulated by use of the toxin, saporin, that can be conjugated to dermorphin, an opioid agonist. Local injection of the conjugate of the toxin and endogenous agonist allows binding of conjugated dermorphin to On cells opioid receptors, subsequent internalisation, access of the toxin to the intracellular space and resultant death of these neurones. This site-specific molecular neurosurgery has been used to study the role of these cells. We combined behavioural, electrophysiological and pharmacological techniques to show that the supraspinal facilitatory drive is essential for neuronal processing of noxious stimuli in normal and neuropathic states, and that descending facilitatory neurones maintain behavioural hypersensitivities to mechanical stimuli after insult and especially during the later stages of nerve injury. This finding, that the origins of the descending pathways are altered in a pathological state, fits well with other studies where lidocaine into the RVM had differential effects on spinal cord neuronal activities. The injection of caused a reduction in the responses of deep dorsal horn neurones to applied stimuli in 64% of normal animals but this was increased to 81% in nerve-injured animals. Whereas responses to noxious inputs were predominantly reduced in the control animals, enhanced reductions in spinal cord activity induced by intra-RVM lidocaine after neuropathy included responses to non-noxious stimuli. This indicates that descending facilitations are the predominant RVM output that impacts the spinal cord in normal animals whilst the influence of these facilitatory descending systems is enhanced after neuropathy, presumably as a result of blockade of facilitatory On cells (Bee and Dickenson 2007, 2008).

## Activation of descending controls

What drives these descending pathways? It is now clearly established that superficial spinal cord neurones, including the NK1 R expressing cells, responding to noxious stimuli project to the parabrachial nucleus and then onwards to higher centres and brainstem nuclei involved in control of the descending pathways.

The increase in pain sensitivity that follows injury is regulated by these superficially located projection neurons in the dorsal horn of the spinal cord that express the neurokinin-1 (NK1) receptor. Pioneering work by Mantyh et al. (1997) showed that after selective ablation of these neurons in rats, there were normal acute pain responses but a loss of hyperalgesia in a number of behavioural assays.

Using neuronal recordings in the spinal cord ablation of these neurones lead to reductions in receptive field size, mechanical and thermal coding and central sensitization of deeper dorsal horn neurones. Then, these changes could be mimicked by pharmacological block of descending serotonergic facilitatory pathways suggesting a pathway from spinal cord lamina I to brain and then back again. With histochemical approaches, changes in the activation of serotonergic neurons in the brainstem was observed as well as a loss of DNIC (Suzuki et al. 2002). Thus, NK1-positive spinal projection neurons, activated by primary afferent input, project to higher brain areas that control spinal excitability—and, therefore, pain sensitivity—primarily through excitatory descending pathways from the brainstem. We then showed the dominance of these descending 5HT excitatory pathways driven by these spinal neurones since the removal of these neurones at the origins lead to a reduction in responsivity despite a concurrent loss of spinal GABA and descending NA controls (Rahman et al. 2008).

Exploring the role of these pathways has lead to the conclusion that there is a balance between noradrenergic inhibitory controls and 5HT facilitations. The former is based on the many studies that have shown powerful inhibitions produced by the activation of the spinal alpha-2 adrenoceptor. The functional roles of this receptor, and the excitatory effects of 5HT that appear to be partly as a result of On cell activity, can be gauged by the use of antagonists that block activity in these pharmacologically defined systems. Spinal application reveals the state of these descending circuits in models of pathophysiological states.

## Altered descending controls

In normal animals, the coding of a wide range of modalities of stimulation has been studied after block of the alpha-2 adrenoceptor and the 5HT3 receptor. It appears that the former inhibitory system modulates lower intensities of mechanical stimuli whereas the excitatory 5HT3 receptor drives responses to higher intensities. Thus, responses to painful stimuli in the normal state are regulated by these descending systems (Suzuki et al. 2004; Bannister and Dickenson 2016a). The human data on an ongoing interaction between brain PAG and spinal cord fits well with this premise (Stroman et al. 2018).

After nerve injury, the situation changes dramatically. There is a loss of the inhibitory NA control and a gain of the facilitatory 5HT3 pathway so that coding of peripheral stimuli at spinal and thalamic levels is now enhanced. With regard to 5HT signalling, after nerve injury there was a novel excitatory effect on lower intensity stimuli as well as thermal signalling but no effect on ongoing activity, further compounded by an appearance of a 5HT2 mediated excitation adding to the function of the 5HT3 receptor (Patel and Dickenson 2018). Behaviourally, depleting 5HT enhanced responses in the later stages of neuropathy, indicating that the neuronal changes are reflected by consequences in the awake animal (Rahman et al. 2006). The noradrenergic control of evoked activities was lost after nerve injury yet was maintained in terms of control of ongoing activity. It is the pathway that fails to be activated since drugs with agonist action of the alpha-2 adrenoceptor maintain, or even have enhanced actions after nerve injury (Bannister and Dickenson 2016b). These results have implications for the use of the Tricyclic antidepressants (TCA) and serotonin-noradrenaline reuptake inhibitors (SNRI) drugs in pain patients since it would appear that agents with NA reuptake actions would be favourable and indeed, SSRIs do considerably less well as analgesics than SNRI and TCAs (Finnerup et al. 2018).

This abnormal descending drive from the RVM impacts upon pain but also the state-dependent inhibitory actions of pregabalin (PGB), used for the treatment of neuropathic pain with actions on spinal alpha-2 delta subunits of calcium channels, influencing transmitter release. The drug is ineffective in the early stages of nerve injury, and its action is prevented by RVM MOR cell ablation but efficacy is restored, by pharmacologically mimicking the descending drive at the spinal level with a 5HT3 receptor agonist (Bee and Dickenson 2008). The mechanism may result from descending 5HT projections impacting upon TRPV1 positive terminals where calcium channels are located, thus enabling PGB actions (Loyd et al. 2011).

Ondansetron, that selectively blocks the 5HT3 receptor, could be considered as a potential treatment but it may not cross the blood brain barrier well, since its anti-emetic action does not require CNS penetration. There are many positive preclinical studies with this drug given spinally (Suzuki et al. 2002; Rahman et al. 2009; Bannister et al. 2015) and a positive study in patients against pain after neuropathy (McCleane et al. 2003). A more nuanced human study was negative, finding no effect on allodynia and ongoing pain (Tuveson et al. 2011). However, the animal data show no role of the 5HT3R on the modulation of these latter modalities in contrast to powerful effects on higher intensity evoked responses thus explaining the discrepant human data (Patel and Dickenson 2018). This is an important issue since there is accumulating evidence that neuropathic pain patients are not likely to have uniform pain control with a particular drug since there are clear subgroups of patients based on their sensory phenotypes (Forstenpointner et al. 2018).

A pain state that could be proposed to involve changes in the central control of the descending systems would be fibromyalgia. Here, the widespread sensitivity to applied stimuli would appear unlikely to result from whole body tissue or nerve injury and the associated symptoms such as fatigue, sensitivity to light and other stimuli, sleep issues and mood changes are indicative of altered emotional and vegetative states which could be driven by limbic brain areas, themselves important in control of the descending pathways. A recent imaging study provided evidence for a balance between descending excitation and inhibition in control subjects but a marked gain of facilitation in FMS patients (Potvin and Marchand 2016; Harper et al. 2018).

## Monitoring activity in descending systems

One method for assessing the functionality of descending controls is through measuring Diffuse Noxious Inhibitory Controls (DNIC) (Yarnitsky 2015). DNIC are a unique form of endogenous inhibitory control, whereby evoked activity of convergent neurons is strongly inhibited by a concurrent noxious stimulus outside of the receptive field (Le Bars et al. 1978a, b; Bannister et al. 2015). Conditioned Pain Modulation (CPM) is the human counterpart of DNIC and can be assessed in the clinic. DNIC cannot be observed in anesthetized animals with spinal cord transection, while CPM is lost in tetraplegics, indicating that both rely on the activation of supraspinal structures and functional descending controls (Le Bars et al. 1978b; Roby-Brami et al. 1987). Many studies in patients have shown that CPM is lost or reduced in pain patients, with the aetiology ranging from neuropathy to migraine (Yarnitsky 2015), indicating that the changes in descending controls are common to many different pain states, unlike the very different peripheral processes exhibited.

## Changes in descending controls in osteoarthritis

Here we will concentrate on pain from osteoarthritis since this common pain condition has been rather less studied than others such as neuropathic pain. Many patients are left with persistent pain even following total joint replacement surgery (Wylde et al. 2011). An alteration in descending controls and supraspinal systems, together with central sensitisation and the possible development of neuropathic pain (Thakur et al. 2014) may be responsible for this persistent pain even though the damaged tissue has been replaced. Indeed, a clinical study using fMRI identified an abnormal descending facilitatory system from the PAG to the spinal cord in patients with hip OA and descriptions of neuropathic pain (Gwilym et al. 2009). An adaptive descending system has also been identified in the MIA model (Rahman et al. 2009; Burnham and Dickenson 2013). First, a 5-HT_3_ receptor antagonist inhibited evoked responses to innocuous stimuli yet did not produce this effect in controls, indicating an alteration in the facilitatory serotonergic system acting at 5-HT_3_ receptors in the spinal cord was modulating low threshold neuronal responses akin to that seen after neuropathy (Rahman et al. 2009). These contrasting serotonergic findings further suggest that the adaptive serotonergic system can switch from an antinociceptive to pronociceptive role depending upon which receptors are activated in the spinal cord. Furthermore, a reduced inhibitory noradrenergic descending system has been demonstrated in the MIA model of osteoarthritis (Burnham and Dickenson 2013). As a subset of OA patients have displayed reduced descending inhibition and an alteration in descending systems from the brainstem, similar alterations in the MIA model suggest this may prove a useful approach through assessing the effectiveness of therapeutics that act to modulate the endogenous descending system (Gwilym et al. 2009; Arendt-Nielsen et al. 2018).

It was observed that early phase MIA animals have a normally functioning DNIC as both concurrent noxious ear and knee pinch produced a significant reduction in mechanically evoked neuronal firing from the foot at similar levels to that observed in saline injected sham controls (Lockwood et al. 2019a). This and previous studies demonstrated that DNIC expression is reliant upon a functioning noradrenergic descending inhibitory system (Bannister et al. 2015; Bannister and Dickenson 2016a; Lockwood et al. 2019a). A previous study reported that early phase MIA animals had a functioning noradrenergic system, as the spinally applied α_2_-adrenergic receptor antagonist atipamezole fully reversed the inhibitory effects produced by the SNRI Milnacipran (Burnham and Dickenson 2013). Furthermore, NSAIDs have been demonstrated to be effective in early phase MIA animals, while late phase MIA animals do not respond indicating the development of central sensitisation (Fernihough et al. 2004). Together these findings suggest that the pain behaviour demonstrated in early phase animals may be a result of peripheral sensitisation and the fully functioning DNIC system may indicate a lack of central changes (Lockwood et al. 2019b).

However, in the late phase animals, there was a complete loss of DNIC as the concurrent noxious ear pinch no longer produced a reduction in mechanically evoked neuronal firing. This is in keeping with indirect data for a reduction in descending noradrenergic inhibitory controls in late phase MIA animals, as atipamezole could not longer fully reverse the inhibitory effects of the SNRI milnacipran (Burnham and Dickenson 2013). Additionally, an enhanced descending serotonergic facilitatory drive acting at 5-HT_3_ receptors in the spinal cord has been demonstrated in late phase MIA animals (Rahman et al. 2009). Interestingly, in a previous study where DNIC was lost in SNL animals, the spinal application of the 5-HT_3_ receptor antagonist ondansetron restored DNIC, indicating that an increased serotonergic facilitatory drive may be contributing to the loss of DNIC (Bannister et al. 2015). Recording the activity of thalamic neurones and manipulating spinal alpha-2 adrenoceptor function reveals a modality specific loss of NA controls of brain activity after nerve injury (Patel et al. 2018). Furthermore, NSAIDs have been demonstrated to be ineffective at relieving hyperalgesia and allodynia in late phase MIA animals, potentially indicating central changes (Fernihough et al. 2004). Therefore, the loss of DNIC in late phase MIA animals observed in this study may be a result of an imbalance in descending controls, specifically a reduced descending inhibitory noradrenergic and enhanced descending facilitatory serotonergic system (Lockwood et al. 2019a).

Remarkably, in some late phase MIA animals a reduction in neuronal firing was observed when the concurrent noxious pinch was placed on the injured knee (Lockwood et al. 2019a). First, this indicates that the DNIC system is not completely abolished and proposes that the activation of the reduced descending noradrenergic system by the conditioning stimulus is no longer sufficient to override the enhanced descending serotonergic facilitatory drive acting at the spinal cord. Overall, the imbalance in descending inhibitory and serotonergic controls is masking the expression of DNIC, this agrees with previous studies demonstrating DNIC can be revealed pharmacologically (Bannister et al. 2015; Bannister and Dickenson 2016a, b). Previous studies using electrophysiological recordings from joint afferents have demonstrated MIA produces a graded sensitisation and increased firing rate (Schuelert and McDougall 2009, 2012). Furthermore, a Nav1.8 channel blocker reduced the firing rate of joint afferents in response to noxious rotation of the joint but had no effect during non-noxious rotation (Schuelert and McDougall 2012). This may indicate that upon noxious conditioning stimulation of the MIA injured knee there is likely an increased firing rate of joint afferents and an increased transduction of nociceptive signals via sodium channels to the dorsal horn. Therefore, the concurrent MIA injured knee pinch may now produce a sufficiently intense enhanced nociceptive barrage, able to activate descending inhibitory controls and produce a small level of neuronal inhibition. Surprisingly, the concurrent contralateral uninjured knee pinch also appeared to produce a significant reduction in neuronal firing in late phase animals, but this was at much lower levels than that observed in early phase animals and sham controls. In human subjects, the intensity of the conditioning stimulus has been shown to be important in eliciting CPM, in line with these studies on DNIC with knee stimulation (Nir et al. 2011; Lockwood et al. 2019b). In good accord with the central mechanisms behind DNIC and CPM, patients with OA show no change in CPM when treated with non steroidal anti-inflammatory drugs that will be acting predominately on peripheral sensitisation (Petersen et al. 2019).

One important aspect of studying chronic pain in animal models is that the findings can be translated to the clinic and inform our understanding of mechanisms and potential therapeutics. As DNIC has a human counterpart, CPM, the DNIC results obtained in the laboratory may hold great clinical relevance and be easily translated to patients. In fact, measuring CPM responses presents a useful tool for assessing the functionality of patients’ descending controls and endogenous inhibitory systems, and can subsequently provide valuable insights on the treatment options (Edwards et al. 2003).

Testing the efficacy of a patient’s CPM system can provide insights into their physiology and likelihood of benefiting from a particular treatment. In fact, in a cohort of patients with painful diabetic neuropathy, baseline CPM was correlated with duloxetine efficacy, such that duloxetine was most effective in patients with a less efficient CPM (Yarnitsky et al. 2012). In addition, a study investigating the development of post-operative chronic pain found that patients with a less efficient CPM before surgery were more susceptible to developing chronic pain after surgery (Yarnitsky et al. 2008) Taken together, these studies indicate that carrying out extensive diagnosis to begin with and testing the functionality of a patient’s CPM system could save time in the long-term, through predicting how likely a patient is to develop chronic pain and which analgesics a patient may respond best to.

Itch can have both inflammatory and neuropathic components in humans and a study of CPM revealed a reduction in CPM in both groups of patients that fits well with the preclinical studies on OA (Pogatzki-Zahn et al. 2019).

Importantly, CPM studies suggest that DNIC and CPM share a similar pharmacology. First, the study demonstrating that patients with a weak CPM benefit from the SNRI duloxetine suggests that similarly to DNIC, noradrenergic and serotonergic signaling pathways subserve CPM (Yarnitsky et al. 2012). In another group of patients, tapentadol, with mu opioid and NRI actions, was able to restore CPM in patients with diabetic neuropathy. In addition, the magnitude of CPM in healthy subjects has been demonstrated to be associated with serotonin transporter gene polymorphisms, indicating that similarly to DNIC in animals, CPM also relies partly on a serotonergic descending system (Lindstedt et al. 2011). Therefore, as the CPM system in humans also relies on monoaminergic signalling, through pharmacologically modulating these systems, the endogenous inhibitory system that CPM utilizes can be restored to provide effective pain relief.

Interestingly, drugs traditionally designed as antidepressants that function by modulating the levels of extracellular noradrenaline and serotonin are often effective at providing pain relief in patients with neuropathy and other chronic pain states (Bomholt et al. 2005; Sindrup et al. 2005). Therefore, the first analgesic target which should be considered in patients with a dysfunctional CPM system is noradrenergic signalling, such as NRIs that prevent the re-uptake of pre-synaptic noradrenaline such that increased levels remain in the synaptic space to carry out inhibitory actions through activating spinal α_2_-adrenergic receptors (Bannister and Dickenson 2016a, b). Tapentadol, which functions partly as an NRI, restores DNIC in late phase MIA animals and also has interactions with pregabalin and so supports this approach (Lockwood and Dickenson 2019). As serotonergic signalling can mediate both inhibitory or facilitatory influences depending upon which receptors become activated in the spinal cord, the consequences of increasing the synaptic availability of serotonin with SSRIs are more complicated. Interestingly, the spinally applied SSRIs, fluoxetine and citalopram, produced antinociception in a model of neuropathy, while systemic selective serotonin reuptake inhibitors (SSRIs) had no effect (Bannister et al. 2017b). Therefore, SSRIs could provide a third or fourth-line therapy for restoring endogenous inhibition in patients, but a more accurate therapeutic approach may be to specifically target the inhibitory serotonergic receptors, such as the 5-HT_7_ receptor which has been shown to mediate the effects of SSRIs applied spinally (Bannister et al. 2017a, b; Lockwood et al. 2019a).

## Brain areas that control descending inhibitory pathways

What are the brain areas that can modulate DNIC? Stroman et al. (2018) using heat stimuli have recently showed that the periaqueductal gray-rostral ventromedial medulla-spinal cord descending modulation pathway, is regulated by input from the hypothalamus, parabrachial nucleus, and nucleus tractus solitarius. The parabrachial nucleus is the first relay in the pathways from the spinal cord lamina I neurones that drive DNIC and represents a portal into the brain whereby threats and other consummatory responses gain access to the amygdala and other areas (Palmiter 2018). In humans, the activation of counterirritation circuits showed that reductions in activity in somatosensory cortex (SI), anterior cingulate cortex and amygdala were produced (Piché et al. 2009). This study also suggested a partly separable control of pain responses and reflexes which is of relevance to preclinical studies where either neuronal or reflex responses are often used to study DNIC. Kappa opioid signalling in the amygdala has been recently reported to be a controller of DNIC in rodents in neuropathic models (Phelps et al. 2019).

A further study on CPM revealed correlations with reduced pain with responses from the primary synapse, the region of the subnucleus reticularis dorsalis (SRD) and the region of the parabrachial nucleus (Youssef et al. 2016a, b). The SRD has been shown in animal studies to be critical for DNIC (Villanueva et al. 1996) but remains a poorly understood link in the pathways from cord to brain and back. Clearly, given the projections of this nucleus to the spinal cord and thalamus, and projections to the nucleus from the somatosensory cortex, a role in modulating pain could be advanced but how does the SRD evoke descending NA controls? In a study in volunteers, where both DNIC and CPM are reduced, greater signal intensity increases during each test stimulus in the presence of the conditioning stimulus compared to test stimuli were seen in the mid-cingulate and dorsolateral prefrontal cortices and increased functional connectivity with the SRD. In contrast, those subjects exhibiting CPM analgesia showed no change in the magnitude of signal intensity increases in these cortical regions or strength of functional connectivity with the SRD (Youssef et al. 2016a, b). Clearly, given the projections of this nucleus to the spinal cord and thalamus, and projections to the nucleus from the somatosensory cortex, a role in modulating pain could be advanced but how does the SRD evoke descending NA controls? It could be that these limbic and cortical areas that control activity in the SRD, in parallel, control the descending pathways including the NA system subserving DNIC. This is supported by data from resting-state fMRI imaging, where patients with chronic neuropathic orofacial pain exhibited increased functional connectivity between the RVM and other brainstem pain-modulatory regions, including the PAG and locus coeruleus. RVM functional connectivity increased onto the first relay of nociceptor afferents, the spinal trigeminal nucleus. Furthermore, the PAG and LC exhibited increased functional connectivity with certain higher brain regions, including the hippocampus, nucleus accumbens, and anterior cingulate cortex, in these patients with chronic pain. Thus, in chronic pain ongoing function within the endogenous pain-modulation network in altered under control from higher centres. The authors suggested that these changes may underlie enhanced descending facilitation of processing at the primary synapse, resulting in increased nociceptive transmission to higher brain centers (Mills et al. 2018). Exactly this has been seen in animal studies where manipulation of the spinal NA inhibitory and 5HT3 excitatory systems in a neuropathic model had a major impact on thalamic neuronal pain processing (Patel et al. 2018; Patel and Dickenson 2018).

Very recently, a preclinical study has shown that activity in the primary somatosensory cortex is able to enhance pain by recruitment of the 5HT3 receptor facilitatory system that we have discussed previously (Tan et al. 2019). A key patient study in OA used imaging and psychophysics (Soni et al. 2019) and consolidated many of the issues that we have covered in this review. Using the PainDETECT questionnaire a group of patients with neuropathic-like pain prior to joint replacement surgery had higher mechanical and cold induced pain near the affected joint. fMRI was used to compare neural activity to that seen in patients without neuropathic-like pain features. Evoked mechanical activity was lower in the rostral anterior cingulate cortex yet higher in the rostral ventromedial medulla (RVM) reflecting functional connectivity between these two areas. These areas are in concordance with a preclinical study on morphine actions on sensory and affective pain measures in rodents (Gomtsian et al. 2018). Most importantly the patient study (Soni et al. 2019) revealed, in keeping with the ideas of a recruitment of the RVM facilitatory (presumably 5HT3 mediated) system in chronic pains (see Bannister and Dickenson 2016a; Bee and Dickenson 2008), that preoperative neuropathic-like pain and higher neural activity in the RVM were associated with moderate-to-severe long-term pain after arthroplasty.

The idea of a widely connected series of brain areas, through as yet, poorly characterised connections, some direct and some indirect, able to modulate and drive the changes in descending facilitations and inhibitions is likely the basis for the recent study showing that alcohol, often used to dull pain, is able to enhance CPM in humans (Horn-Hofmann et al. 2019).

## Conclusions

Overall, the similarities in the descending systems and in particular, DNIC and CPM systems indicate that studies on the functionality of brain systems that communicate with the spinal cord provide promising potential for forward translation from preclinical studies to patients. But descending control changes are only part of the pain transmission changes that accompany chronic pain. In a study of patients with chronic pain, CPM and central sensitisation (CS) were assessed. Patients with both impaired CPM and enhanced CS had the most pain and greatest pain areas—these formed 21% of the sample, and a further 37% with considerable hyperalgesias had reduced CPM showing that attenuated descending inhibitions are common in persistent pain patients (Vaegter and Graven-Nielsen 2016).

It is becoming clear that the close functional, pharmacological and anatomical parallels, and thus the potential to translate back and forward from patients and animals allows descending controls to take a major position in the understanding of pain modulatory mechanisms. The ability of conditioned pain modulation in patients, based on the pharmacological studies in preclinical groups on DNIC in animals, to predict the actions of drugs with noradrenergic mechanisms is another step forward in attempts to produce precision medicine in patients with neuropathic pain. This account provides a preclinical basis for studies in patients with severe osteoarthritis using the same approaches.
